# Effect of SDF-1 and CXCR4 gene variants on the development of diabetic kidney disease

**DOI:** 10.7150/ijms.103186

**Published:** 2024-10-28

**Authors:** Ke-Hsin Ting, Po-Jen Yang, Shih-Chi Su, Po-Yu Tsai, Shun-Fa Yang

**Affiliations:** 1 Division of Cardiology, Department of Internal Medicine, Changhua Christian Hospital, Yunlin Branch, Yunlin, Taiwan.; 2 Department of Nursing, Hungkuang University, Taichung, Taiwan.; 3 Department of Post-Baccalaureate Medicine, College of Medicine, National Chung Hsing University, Taichung, Taiwan.; 4 Institute of Medicine, Chung Shan Medical University, Taichung, Taiwan.; 5 School of Medicine, Chung Shan Medical University, Taichung, Taiwan.; 6 Department of Family and Community Medicine, Chung Shan Medical University Hospital, Taichung, Taiwan.; 7 Whole-Genome Research Core Laboratory of Human Diseases, Chang Gung Memorial Hospital, Keelung, Taiwan.; 8 Department of Medical Biotechnology and Laboratory Science, College of Medicine, Chang Gung University, Taoyuan, Taiwan.; 9 Division of Nephrology, Department of Internal Medicine, Chung Shan Medical University Hospital, Taichung, Taiwan.; 10 Department of Medical Research, Chung Shan Medical University Hospital, Taichung, Taiwan.

**Keywords:** SDF-1, CXCR4, gene polymorphism, diabetic kidney disease

## Abstract

Diabetic kidney disease (DKD) is the gradual loss of renal function occurring in patients with diabetes. Stromal cell-derived factor-1 (SDF-1, encoded by *SDF-1* gene) is a chemokine that binds to its receptor, CXCR4, to mediate many aspects of renal biology. To test the potential impact of *SDF-1/CXCR4* gene variations on the risk for DKD, single-nucleotide polymorphisms (SNPs) of* SDF-1/CXCR4* genes were genotyped in 388 DKD patients and 335 DKD-free diabetic controls. Among 6 SNPs examined, we demonstrated that rs1801157 of *SDF-1* gene was associated with an increased risk for DKD (GA vs GG, AOR=2.252, *p*=0.035; GA+AA vs GG, AOR=2.156, *p*=0.036). Further stratification revealed that the correlation of rs1801157 with DKD was particularly detected in diabetic patients with early CKD but not in those with severe renal impairment. Instead, another SNP of *SDF-1* gene, rs266085, was found in association with the advanced form of DKD (TC vs TT, AOR=2.106, *p*=0.027; TC+CC vs TT, AOR=2.130, *p*=0.019), indicating differential impacts of *SDF-1* gene polymorphisms on the progressive loss of renal function in diabetic patients. Moreover, preliminary survey of public gene expression datasets showed that rs1801157 and rs266085 modulated *SDF-1* expression in many human tissues, and SDF-1/CXCR4 levels were elevated in renal tissues of DKD patients. These data suggest that allele-specific expression of *SDF-1* gene may influence DKD progression.

## Introduction

As the microvascular damages to the kidney frequently arise in diabetes, diabetic kidney disease (DKD), a hallmark comorbidity of diabetes that occurs in roughly 20-50% of patients with type 2 diabetes mellitus (T2DM) 1, has been believed to stem from a complex interplay of metabolic, inflammatory, hemodynamic, and fibrotic abnormalities 2. These dysfunctions, comprising massive elimination of metabolic products derived from distorted glucose catabolism 3, perturbation of the renin-angiotensin-aldosterone system (RAAS) 4, and modulation of various signaling pathways associated with renal fibrosis 5, 6, toxicity of reactive oxygen species 7, 8, complement cascade 9, and inflammatory activity 10, 11, to a large extent account for the pathophysiology of DKD, resulting in renal impairment. Diverse risk factors for DKD are known and conceptually classified as susceptibility (e.g., age, gender, and genetic inheritance), initiation (e.g., hyperglycemia and acute kidney injury), and progression factors (e.g., obesity, hypertension, and diet) 12. Among these established risks, hyperglycemia, obesity, and hypertension are conceivably modifiable via appropriate diabetes management 13. This complex nature of disease etiology contributes to a high variation of DKD epidemiology and clinical outcomes, thereby urging us for the identification of new targets or manipulable parameters to ameliorate the prevention and treatment of DKD.

Cumulative evidence has clearly linked genetic inheritance to the development of both diabetes and its complications 14. Moreover, a family history of cardiovascular conditions in diabetic individuals was found to be associated with an increased risk for DKD 15, 16, highlighting an impact of inherited factors on developing DKD in DM cases. Until now, genome-wide screening has uncovered a myriad of genetic variants that confer the susceptibility to diabetes and DKD 17-21. These genes represent the genetic landscape of diabetes and shed light on the understanding of DKD pathogenesis, notably in the glycemic control, albuminuria, and renal function decline in distinct racial groups 22, 23. Yet, the architecture of DKD predisposing factors is of great heterogeneity and only partly explains why some individuals suffer from renal diseases and some preserve kidney function 24. Thus, further dissecting genetic background of DKD may provide additional biomarkers for the improvement of disease diagnosis and management.

Stromal cell-derived factor 1 (SDF-1), also named CXCL12, was initially identified as a member of CXC chemokine family secreted by bone marrow stromal cells 25, 26. It is known that SDF-1 binds to its cognate receptor, CXC chemokine receptor 4 (CXCR4), to mediate diverse biological effects, such as the induction of cell adhesion, proliferation, motility, chemotactic activity, and angiogenic responses 27, 28. In the kidney, SDF-1 was shown to be expressed in the stromal cells and podocytes of mature glomeruli to control nephrogenesis and the development of renal vasculature 29, 30. As renal SDF-1 has been demonstrated to play a key role in kidney repair 31, a crucial involvement of SDF-1 signal in the pathogenesis of many kidney diseases or renal complications of diabetes was proposed 32. Moreover, blockage of SDF-1/CXCR4 axis in the rodent models of diabetes caused podocyte loss and promoted mesangial expansion and tubular epithelial cell death, thereby leading to albuminuria and glomerulosclerosis 33, 34. These observations indicate a connection between SDF-1/CXCR4 axis and renal health in diabetic patients. Additionally, there have been significant efforts to explore whether single nucleotide polymorphisms (SNPs) of *SDF-1* gene (encoding SDF-1) influence the susceptibility to acquired immunodeficiency syndrome (AIDS) 35, chronic myeloproliferative disease 36 and many forms of malignancies 37. To date, the association of *SDF-1* gene polymorphisms with the development of DKD remains elusive, as an effect of *SDF-1* gene variations on an ocular complication of diabetes, diabetic retinopathy, has been reported 38. In this case-control study, we attempted to test the potential impact of *SDF-1/CXCR4* gene polymorphisms on the risk for DKD.

## Materials and methods

### Study cohorts

In this study, 388 DKD cases and 335 diabetic patients with normal kidney function [estimated by glomerular filtration rate (GFR)] were enrolled and approval by the institutional review board (CSMUH No: CS2-22190) in Chung Shan Medical University Hospital, Taichung, Taiwan. Kidney disease was diagnosed as the sign of albuminuria or an estimated GFR (eGFR) of less than 60 mL/min/1.73 m2 from two individual visits. To exploring the disease severity, we stratified DKD cases into early CKD (n=308, CKD stage 1-3; with an eGFR ≥ 30) and pre-ESRD (n=80, CKD stage 4-5; with an eGFR < 30). Demographic and laboratory data regarding age, gender, diabetic condition, hyperlipidemic status, and renal function were obtained.

### Genotyping

A total of six SNPs, including four from the *SDF-1* gene (rs1801157, rs2297630, rs2839693, and rs266085) and two from the *CXCR4* gene (rs2228014 and rs6430612) were examined based on their connection with the susceptibility to various diseases 37-41. Genomic DNA of the whole blood samples was isolated by using QIAamp DNA Blood Mini kit (Qiagen, Valencia, CA, USA). Biallelic discrimination for rs1801157 (assay ID: C_3223115_10, rs2297630 (assay ID: C_3223122_1), rs2839693 (assay ID: C_31777299_10), rs266085 (assay ID: C_1033724_30), rs2228014 (assay ID: C_27378716_10) and rs6430612 (assay ID: C_30721949_10) SNPs was carried out through the TaqMan assay (Applied Biosystems, Foster City, CA, USA), and genotypes were determined by SDS version 3.0 software.

### Statistical analysis

Hardy-Weinberg equilibrium for six selected SNPs was assessed by using a χ^2^ goodness-of-fit approach. Demographic and laboratory data between cases and controls were compared with the Mann-Whitney U test. Association of polymorphic alleles with the risk and severity of DKD was evaluated by multiple logistic regression analyses combined with the adjustment for potential confounders (age, the duration of diabetes, HbA1c, systolic blood pressure, serum creatinine levels, glomerular filtration rate, HDL cholesterol levels, LDL cholesterol levels, triglycerides levels, TC/HDL ratio, microalbumin and UACR). Differences in *SDF-1* expression among genotypic groups from the Genotype-Tissue Expression (GTEx) database 42 were calculated with one-way ANOVA. Gene expression of SDF-1 and CXCR4 was retrieved from the Gene Expression Omnibus repository (GSE30122) 43 and compared by t-test. A *p* value of <0.05 was considered statistically significant.

## Results

### Baseline characteristics of study cohorts

To examine the potential effect of *SDF-1/CXCR4* gene variants on the risk for DKD, 388 DKD cases were recruited and compared with 335 CKD-free controls. The baseline characteristics of two study groups were evaluated (**Table 1**). Differences in the age but not the gender were detected between cases and controls. The mean of age in DKD cases and controls was 62.74 and 59.13 (years), respectively. Besides common indications of kidney function decline (impaired glomerular filtration rate and elevation of urinal albumin and serum creatinine), an increase in the duration of diabetic conditions and severity of hyperglycemia (determined by HbA1c values) was seen in the case group, as compared with controls. Furthermore, several indications of cardiovascular diseases, such as systolic blood pressure and ratio of total cholesterol to high-density lipoprotein cholesterol, were elevated in DKD patients in comparison with CKD-free controls.

### Effect of SDF-1/CXCR4 gene polymorphisms on the risk for DKD

To test the influence of *SDF-1/CXCR4* gene variations on the susceptibility to DKD, genotypes of four SNPs from the *SDF-1* gene (rs1801157, rs2297630, rs2839693, and rs266085) and of two SNPs from the *CXCR4* gene (rs2228014 and rs6430612) were surveyed in our cohorts. For all six SNPs tested, no deviation (*p*>0.05) from Hardy-Weinberg equilibrium in both study cohorts was seen. Of note, we observed that diabetic individuals who are heterozygous for the minor allele (A) of *SDF-1* rs1801157 (GA; AOR, 2.252; 95% CI, 1.060-4.741; *p*=0.035) are more frequently to develop renal complications (**Table 2**), while diabetic patients homozygous for the major allele (G) of rs1801157 (GG) are used as the reference in additive model. In addition, DM patients who carry at least one minor allele (A) of *SDF-1* rs1801157 (GA and AA; AOR, 2.156; 95% CI, 1.051-4.424; *p*=0.036) are more commonly suffering from CKD than are those homozygotes for the major allele (GG) in dominant model. Yet, for two SNPs of the *CXCR4* gene tested, no interaction with the risk for DKD was detected from our study cohorts (**Table 2**). However, there are no significant correlations noted in allele model (**Table 3**). These data indicate that *SDF-1* rs1801157 genotypes confer the predisposition to renal complications in diabetic patients.

### Differential effects of SDF-1/CXCR4 gene polymorphisms on the disease progression of DKD

Since a genetic predisposition of DKD was observed in our cohorts, we subsequently performed stratification analyses to explore whether specific genotypes of *SDF-1/CXCR4* genes are associated with the progression of renal impairment. By stratifying DKD cases into two severity groups (early CKD and pre-ESRD), we found that the correlation of *SDF-1* rs1801157 with DKD was particularly detected in diabetic patients with early CKD (GA vs GG, AOR, 2.198; 95% CI, 1.036-4.663, *p*=0.040; GA+AA vs GG, AOR, 2.116; 95% CI, 1.029-4.353, *p*=0.042) in additive model and dominant model (**Table 4**). However, this genetic association was not observed in diabetic subjects with advanced CKD (the pre-ESRD group) (**Table 5**). Instead, specific genotypes of another SNP of *SDF-1* gene, rs266085, were associated with advanced DKD (the pre-ESRD) (TC vs TT, AOR, 2.106; 95% CI, 1.090-4.069, *p*=0.027; TC+CC vs TT, AOR, 2.130; 95% CI, 1.130-4.014, *p*=0.019) in additive model and dominant model (**Table 5**). These results indicate differential impacts of *SDF-1* gene polymorphisms on the progressive loss of kidney function in DM patients.

### Functional insight of rs1801157 and rs266085 in DKD

**Figure 1:**
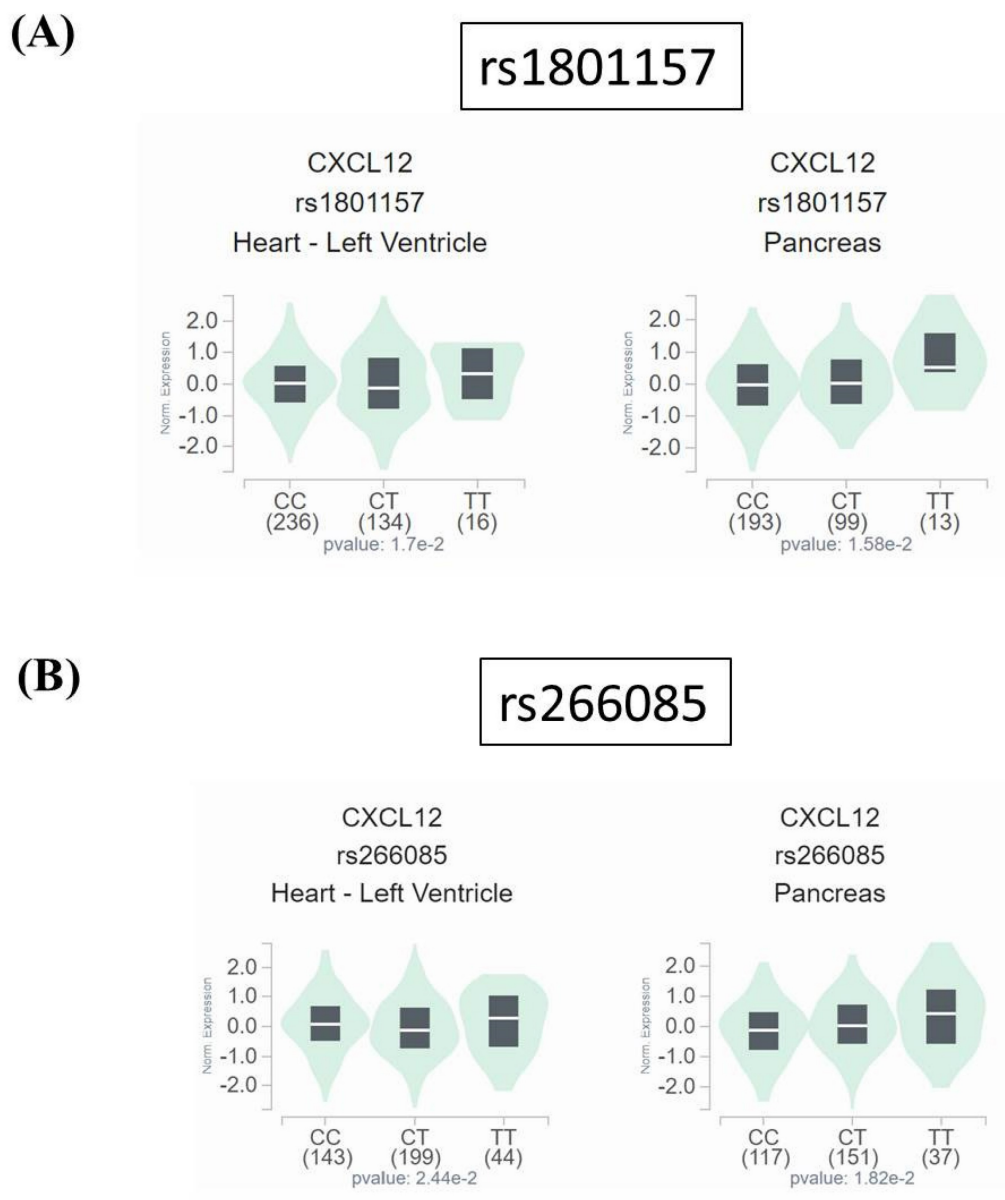
** Impact of rs1801157 and rs266085 genotypes on SDF-1 expression.** Comparisons of SDF-1 (CXCL12) expression among (A) rs1801157 and (B) rs266085 genotypic groups in representative normal tissues based on data from the GTEx portal. *p* values were calculated among groups by one-way ANOVA.

**Figure 2:**
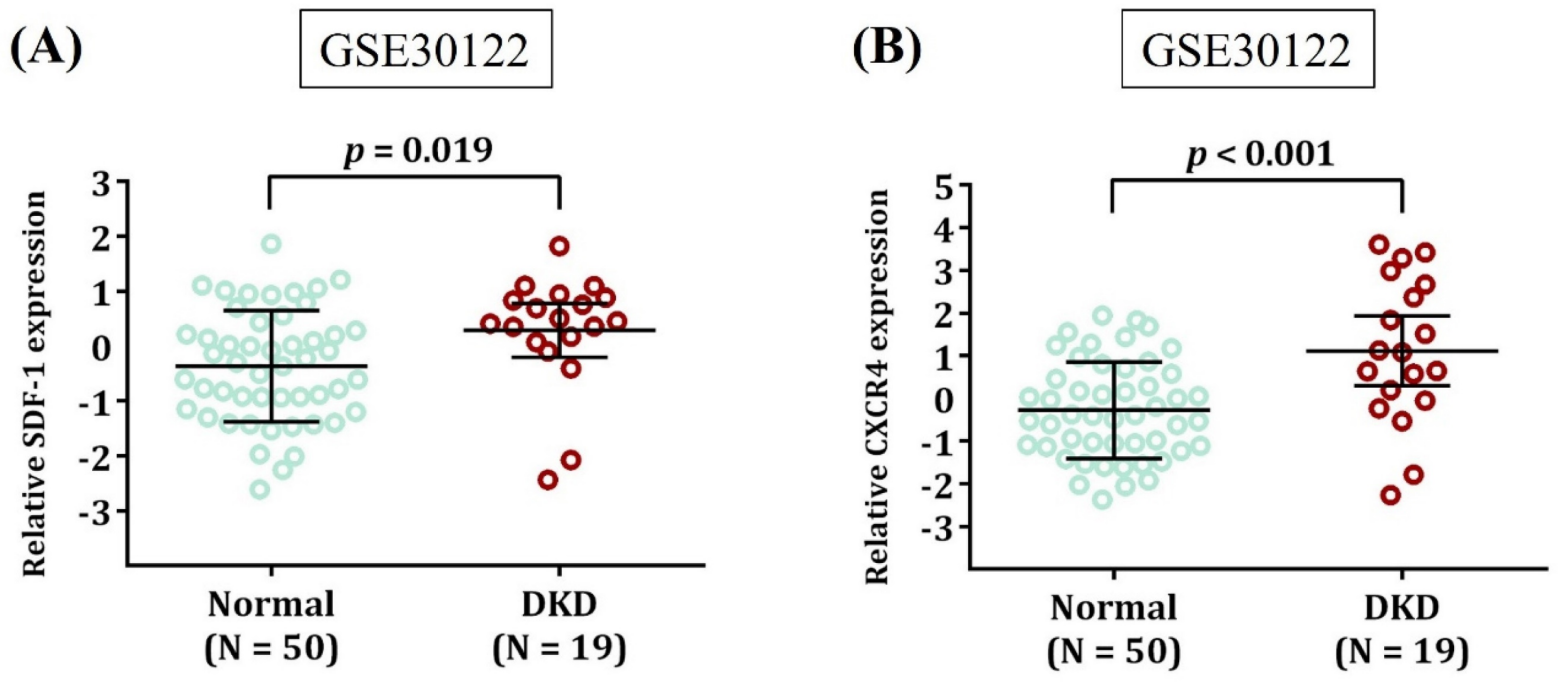
** Expression levels of SDF-1 and CXCR4 are increased in DKD.** Comparison of SDF-1 **(A)** and CXCR4 expression **(B)** in the renal tissues between DKD patients and healthy donors (Normal). Expression data were retrieved from Gene Expression Omnibus repository (GSE30122). The total number of samples is given in parentheses. *p* values are calculated with Student's *t* test.

Since neither rs1801157 nor rs266085 is located on the coding region of *SDF-1* gene, we also surveyed public datasets to obtain a possible clue for the function of these two DKD-associated alleles. We demonstrated fluctuations of *SDF-1* expression in the heart and pancreas among different genotypic groups of rs1801157 and rs266085 in the Genotype-Tissue Expression (GTEx) database (**Figure 1**). In addition, through analyzing a transcriptomic dataset in Gene Expression Omnibus repository (GSE30122) 43, a significant increase in expression levels of SDF-1 and its receptor, CXCR4, was noted in renal tissues of DKD patients in comparison with that of healthy donors (**Figure 2**). There data imply that alteration in allele-specific *SDF-1* expression might influence the disease progression of DKD.

## Discussion

A body of emerging evidence has pointed out that the complex etiology of DKD is controlled by an interplay of genetic and acquired parameters. Here, by using a candidate gene approach, we demonstrated an association of *SDF-1* rs1801157 with the initiation of DKD. In addition, genotypes of *SDF-1* rs266085 were correlated with the progression into the advanced form of DKD, unveiling a differential effect of *SDF-1* gene variations on orchestrating the disease course of DKD.

Genetic variations in *SDF-1* gene have configured intricate patterns of disease susceptibility. Among these disease-associated alleles of *SDF-1* gene, one SNP located in the 3' untranslated region (3'UTR), rs1801157, is the best-studied polymorphism. It has been demonstrated that rs1801157 is correlated with many clinical manifestations of human immunodeficiency virus (HIV) infection, outcomes of liver transplantation, age-at-onset of diabetes, and risks for hematologic malignancies and solid tumors 44. In this study, we extend the list of its involvements in pathological conditions to include renal complications of diabetes. Moreover, rs1801157 gene polymorphism is associated with susceptibility to adverse long-term allograft outcomes in non-diabetic kidney transplant recipients 45. The functional relevance of rs1801157 variants was originally accounted for by induction of SDF-1 levels 35. However, opposite findings that carriers homozygous for the minor allele (A) of rs1801157 was associated with low plasma SDF-1 levels have been reported 46, 47, while other studies documented that there was no difference in the SDF-1 production by the presence of rs1801157 polymorphisms 48, 49. Such controversies might be attributed to the frequency and structure of unique haplotypes in different ethnic groups, as additional SNPs in high linkage disequilibrium with rs1801157, instead of rs1801157 itself, were found to mediate differential transcription levels 50. These observations collectively indicate that rs1801157 likely contributes to allele-specific expression of *SDF-1* gene to render a functional impact.

As a matter of fact, expression levels of SDF-1 are crucial for renal development and exert modulatory effects on the course of kidney diseases under different etiologic settings by binding to its cognate receptor, CXCR4 32. Expression of SDF-1 by stromal cells or podocytes acts on endothelial cells to regulate vascular and glomerular development in the kidney 30. In a mouse model of diabetes, aberrant expression of SDF-1 by glomerular podocytes augmented proteinuria and glomerulosclerosis, while the use of a specific inhibitor of SDF-1, NOX-A12, corrected glomerulosclerosis, enhanced the number of podocytes, maintained the peritubular vasculature and delayed the onset of albuminuria 33. On the contrary, reduction of endothelial SDF-1 was accompanied by proteinuria, elevated oxidative stress, podocyte foot process effacement and augmented glomerular size in a rat model of obesity 51. Also, SDF-1 was proposed to participate in promotion of renal fibrosis, which is believed to be the final common pathogenic mechanism leading to CKD and ESRD 52. In addition to the involvement in the progressive loss of renal function, robust upregulation of SDF-1 after ischemia/reperfusion-induced acute kidney injury contributed to homing and migration of CXCR4-positive cells toward the injured kidney, governing renal regeneration and repair 31. These results, together with our findings, support a conjecture that fluctuations in SDF-1 levels derived from gene polymorphisms affect the susceptibility to DKD in diabetic patients.

Intriguingly, we exhibited an association of an intronic SNP of *SDF-1* gene, rs266085, with the risk for the advanced form of DKD. It has been reported that rs266085 was correlated with the susceptibility to cervical carcinoma, likely via production of distinct *SDF-1* splice variants 53. To date, at least six human SDF-1 isoforms derived from alternative splicing events have been identified (SDF-1α, SDF-1β, SDF-1γ, SDF-1δ, SDF-1ε, and SDF-1Φ) 54, 55 and subjected to different proteolytic processing 56, thus explaining functional diversity. All these *SDF-1* splice variants share the same first three exons but contain different fourth exons 55. Although rs266085 is located in the third intron between exon 2 and 3 of the *SDF-1* gene, another intronic SNP located between exon 3 and 4 of the *SDF-1* gene, rs266087, was in perfect linkage disequilibrium with rs266085 53. It is possible that rs266087 (or others variants on the same common haplotype) affected the relative accumulation of different SDF-1 isoforms 50. In addition to generation of different SDF-1 isoforms, the same study indicated that specific haplotypes containing the minor allele of rs266085 were associated with strong SDF-1 induction, implicating a potential role of rs266085 in allele-specific expression of different *SDF-1* splice variants.

As the exclusive receptor for SDF-1, CXCR4 has been implicated as a crucial mediator of renal regeneration and kidney diseases 57. However, for two SNPs (rs2228014 and rs6430612) of the *CXCR4* gene tested, no significant association with the risk for DKD was observed from our cohorts. As a relatively low frequency (<10%) for the alternative allele of rs6430612 was detected in our study groups, rs2228014 polymorphism is more common in our survey. To date, many case-control studies aiming to investigate the association of *CXCR4* rs2228014 with disease susceptibility have yielded conflicting results 58-64, which may be accounted for by insufficient sample size of individual study, different distributions of cases or controls, different disease pathology, and various methodologies. Determining the relationship between *CXCR4* variants and DKD risks will require further investigation with a greater sample size and subgroup analyses.

In our survey, we detected an association between* SDF-1* gene variants and the development of DKD. However, there are several limitations to this investigation. One potential issue is that the diverse comorbidities of diabetes (e.g. ocular, cutaneous, neurological, cardiovascular, and muscular conditions) and their inherent genetic components likely result in a different finding concerning the impact of *SDF-1/CXCR4* gene variations with DKD. Moreover, causal expression quantitative trait loci were highly enriched in 3'UTR 65, yet we did not test whether rs1801157 variants orchestrate *SDF-1* transcription in our own cohort or related kidney cell types, such as podocytes, renal stromal, and endothelial cells. Also unavailable are the data to support a link of rs266085 to alternative splicing, as well as to dissect an involvement of *SDF-1* splice variants in DKD pathogenesis through functional validation. Additionally, recent multi-ethnic investigations indicated an ethnicity-specific involvement of *SDF-1* variants in disease susceptibility 37, 66, suggesting the presence of variations in the frequency of particular *SDF-1* alleles among different ethnic cohorts. Thus, the genetic impact observed in our study may be constrained to unique populations unless a replication cohort with other ethnicities was investigated.

In conclusion, our data unveiled an impact of *SDF-1* gene variations on acting as a gatekeeper during the disease course of DKD. This genetic association likely connects allele-specific expression of *SDF-1* splice variants to the aggravation of renal impairment in diabetic subjects.

## Figures and Tables

**Table 1:** Clinical and laboratory characteristics of diabetic patients with/without kidney diseases.

Variable	Non-diabetic kidney disease (N=335)	Diabetic kidney disease (N=388)	*p* value
Age (years)	59.13 ± 11.01	62.74 ± 10.96	<0.001
Male gender [n (%)]	173 (51.6%)	217 (55.9%)	0.249
Duration of diabetes (years)	9.10 ± 6.60	11.55 ± 8.04	<0.001
HbA1c [% (mmol/mol)]	6.95 ± 0.96	7.40 ± 1.35	<0.001
Body mass index [kg/m^2^]	25.78 ± 4.13	26.38 ± 4.43	0.064
Systolic blood pressure [mmHg]	133.16 ± 15.58	138.85 ± 16.27	<0.001
Diastolic blood pressure [mm Hg]	75.76 ± 11.19	76.62 ± 11.59	0.311
Serum creatinine [mg/dL]	0.79 ± 0.19	1.43 ± 1.56	<0.001
Glomerular filtration rate [ml/min]	85.94 ± 26.43	61.23 ± 30.38	<0.001
Total cholesterol [mmol/L]	164.02 ± 36.97	160.72 ± 50.54	0.327
HDL cholesterol [μmol/L]	47.12 ± 12.77	43.94 ± 13.09	0.001
LDL cholesterol [μmol/L]	89.58 ± 26.73	83.87 ± 32.30	0.011
Triglycerides, [μmol/L]	134.86 ± 115.90	156.01 ± 173.10	0.060
TC/HDL ratio	3.66 ± 1.12	3.96 ± 2.37	0.040
Microalbumin (mg/dL)	1.12 ± 1.07	48.68 ± 103.33	<0.001
UACR (mg/g)	9.97 ± 7.09	613.81 ± 1457.35	<0.001

**Table 2:** Association between SDF-1/CXCR4 genotypes and diabetic kidney disease.

Variable	Non-diabetic kidney disease (N=335)	diabetic kidney disease (N=388)	AOR (95% CI)	*p* value
**SDF-1** **rs1801157**				
**Additive model**				
GG	175 (52.2%)	176 (45.4%)	1.000 (reference)	
GA	139 (41.5%)	180 (46.4%)	2.242 (1.060-4.741)	*p*=0.035
AA	21 (6.3%)	32 (8.2%)	1.741 (0.441-6.875)	*p*=0.429
**Dominant model**				
GG	175 (52.2%)	176 (45.4%)	1.000 (reference)	
GA+AA	160 (47.8%)	212 (54.6%)	2.156 (1.051-4.424)	*p*=0.036
**SDF-1** **rs2297630**				
**Additive model**				
GG	267 (79.7%)	303 (78.1%)	1.000 (reference)	
GA	60 (17.9%)	75 (19.3%)	0.583 (0.211-1.613)	*p*=0.299
AA	8 (2.4%)	10 (2.6%)	0.571 (0.052-6.274)	*p*=0.647
**Dominant model**				
GG	267 (79.7%)	303 (78.1%)	1.000 (reference)	
GA+AA	68 (20.3%)	85 (21.9%)	0.581 (0.224-1.509)	*p*=0.265
**SDF-1** **rs2839693**				
**Additive model**				
CC	263 (78.5%)	296 (76.3%)	1.000 (reference)	
CT	68 (20.3%)	87 (22.4%)	1.832 (0.769-4.367)	*p*=0.172
TT	4 (1.2%)	5 (1.3%)	0.597 (0.029-12.144)	*p*=0.737
**Dominant model**				
CC	263 (78.5%)	296 (76.3%)	1.000 (reference)	
CT+TT	72 (21.5%)	92 (23.7%)	1.712 (0.726-4.036)	*p*=0.219
**SDF-1** **rs266085**				
**Additive model**				
TT	119 (35.5%)	123 (31.7%)	1.000 (reference)	
TC	166 (49.6%)	193 (49.7%)	0.689 (0.319-1.488)	*p*=0.343
CC	50 (14.9%)	72 (18.6%)	1.150 (0.402-3.285)	*p*=0.794
**Dominant model**				
TT	119 (35.5%)	123 (31.7%)	1.000 (reference)	
TC+CC	216 (64.5%)	265 (68.3%)	0.786 (0.384-1.608)	*p*=0.509
**CXCR4** **rs2228014**				
**Additive model**				
CC	257 (76.7%)	295 (76.0%)	1.000 (reference)	
CT	70 (20.9%)	84 (21.6%)	1.581 (0.667-3.745)	*p*=0.298
TT	8 (2.4%)	9 (2.4%)	1.845 (0.186-18.342)	*p*=0.601
**Dominant model**				
CC	257 (76.7%)	295 (76.0%)	1.000 (reference)	
CT+TT	78 (23.3%)	93 (24.0%)	1.606 (0.703-3.671)	*p*=0.261
**CXCR4** **rs6430612**				
**Additive model**				
CC	310 (92.5%)	353 (91.0%)	1.000 (reference)	
CT	25 (7.5%)	35 (9.0%)	1.233 (0.370-4.110)	*p*=0.734
TT	0 (0.0%)	0 (0.0%)	---	---
**Dominant model**				
CC	310 (92.5%)	353 (91.0%)	1.000 (reference)	
CT+TT	25 (7.5%)	35 (9.0%)	1.233 (0.370-4.110)	*p*=0.734

The adjusted odds ratio (AOR) with their 95% confidence intervals were estimated by multiple logistic regression models.

**Table 3:** Association between SDF-1/CXCR4 genotypes and diabetic kidney disease with genetic allele model.

Variable	Non-diabetic kidney disease (N=670)	diabetic kidney disease (N=776)	OR (95% CI)	*p* value
**SDF-1** **rs1801157**				
**Allele model**				
G	489 (73.0%)	532 (68.6%)	1.000 (reference)	
A	181 (27.0%)	244 (31.4%)	1.239 (0.986-1.557)	*p*=0.065
**SDF-1** **rs2297630**				
**Allele model**				
G	594 (88.7%)	681 (87.8%)	1.000 (reference)	
A	76 (11.3%)	95 (12.2%)	1.090 (0.791-1.503)	*p*=0.598
**SDF-1** **rs2839693**				
**Allele model**				
C	594 (88.7%)	679 (87.5%)	1.000 (reference)	
T	76 (11.3%)	97 (12.5%)	1.117 (0.811-1.537)	*p*=0.499
**SDF-1** **rs266085**				
**Allele model**				
T	404 (60.3%)	439 (56.6%)	1.000 (reference)	
C	266 (39.7%)	337 (43.4%)	1.166 (0.945-1.438)	*p*=0.152
**CXCR4** **rs2228014**				
**Allele model**				
C	584 (87.2%)	674 (86.9%)	1.000 (reference)	
T	86 (12.8%)	102 (13.1%)	1.028 (0.756-1.398)	*p*=0.862
**CXCR4** **rs6430612**				
**Allele model**				
C	645 (96.3%)	741 (95.5%)	1.000 (reference)	
T	25 (3.7%)	35 (4.5%)	1.219 (0.722-2.058)	*p*=0.459

**Table 4:** Association between SDF-1/CXCR4 genotypes and early diabetic kidney disease.

Variable	Non-diabetic kidney disease (N=335)	Early CKD (N=308)	AOR (95% CI)	*p* value
**SDF-1** **rs1801157**				
**Additive model**				
GG	175 (52.2%)	137 (44.5%)	1.000 (reference)	
GA	139 (41.5%)	143 (46.4%)	2.198 (1.036-4.663)	*p*=0.040
AA	21 (6.3%)	28 (9.1%)	1.721 (0.438-6.758)	*p*=0.436
**Dominant model**				
GG	175 (52.2%)	137 (44.5%)	1.000 (reference)	
GA+AA	160 (47.8%)	171 (55.5%)	2.116 (1.029-4.353)	*p*=0.042
**SDF-1** **rs2297630**				
**Additive model**				
GG	267 (79.7%)	238 (77.3%)	1.000 (reference)	
GA	60 (17.9%)	62 (20.1%)	0.593 (0.215-1.633)	*p*=0.312
AA	8 (2.4%)	8 (2.6%)	0.614 (0.056-6.715)	*p*=0.689
**Dominant model**				
GG	267 (79.7%)	238 (77.3%)	1.000 (reference)	
GA+AA	68 (20.3%)	70 (22.7%)	0.595 (0.230-1.544)	*p*=0.286
**SDF-1** **rs2839693**				
**Additive model**				
CC	263 (78.5%)	238 (77.3%)	1.000 (reference)	
CT	68 (20.3%)	65 (21.1%)	1.846 (0.774-4.402)	*p*=0.167
TT	4 (1.2%)	5 (1.6%)	0.613 (0.032-11.899)	*p*=0.746
**Dominant model**				
CC	263 (78.5%)	238 (77.3%)	1.000 (reference)	
CT+TT	72 (21.5%)	70 (22.7%)	1.724 (0.731-4.066)	*p*=0.213
**SDF-1** **rs266085**				
**Additive model**				
TT	119 (35.5%)	105 (34.1%)	1.000 (reference)	
TC	166 (49.6%)	146 (47.4%)	0.693 (0.321-1.497)	*p*=0.350
CC	50 (14.9%)	57 (18.5%)	1.174 (0.410-3.362)	*p*=0.764
**Dominant model**				
TT	119 (35.5%)	105 (34.1%)	1.000 (reference)	
TC+CC	216 (64.5%)	203 (65.9%)	0.793 (0.387-1.622)	*p*=0.525
**CXCR4** **rs2228014**				
**Additive model**				
CC	257 (76.7%)	232 (75.3%)	1.000 (reference)	
CT	70 (20.9%)	68 (22.1%)	1.591 (0.674-3.758)	*p*=0.289
TT	8 (2.4%)	8 (2.6%)	1.858 (0.196-17.595)	*p*=0.589
**Dominant model**				
CC	257 (76.7%)	232 (75.3%)	1.000 (reference)	
CT+TT	78 (23.3%)	76 (24.7%)	1.618 (0.711-3.682)	*p*=0.252
**CXCR4** **rs6430612**				
**Additive model**				
CC	310 (92.5%)	276 (89.6%)	1.000 (reference)	
CT	25 (7.5%)	32 (10.4%)	1.173 (0.344-4.001)	*p*=0.799
TT	0 (0.0%)	0 (0.0%)	---	---
**Dominant model**				
CC	310 (92.5%)	276 (89.6%)	1.000 (reference)	
CT+TT	25 (7.5%)	32 (10.4%)	1.173 (0.344-4.001)	*p*=0.799

The adjusted odds ratio (AOR) with their 95% confidence intervals were estimated by multiple logistic regression models.

**Table 5:** Association between SDF-1/CXCR4 genotypes and Pre-ESRD disease.

Variable	Non-diabetic kidney disease (N=335)	Pre-ESRD (N=80)	AOR (95% CI)	*p* value
**SDF-1** **rs1801157**				
**Additive model**				
GG	175 (52.2%)	39 (48.7%)	1.000 (reference)	
GA	139 (41.5%)	37 (46.3%)	1.163 (0.328-4.123)	*p*=0.815
AA	21 (6.3%)	4 (5.0%)	0.715 (0.193-2.640)	*p*=0.614
**Dominant model**				
GG	175 (52.2%)	39 (48.7%)	1.000 (reference)	
GA+AA	160 (47.8%)	41 (51.3%)	0.964 (0.276-3.364)	*p*=0.954
**SDF-1** **rs2297630**				
**Additive model**				
GG	267 (79.7%)	65 (81.3%)	1.000 (reference)	
GA	60 (17.9%)	13 (16.3%)	0.463 (0.054-3.987)	*p*=0.483
AA	8 (2.4%)	2 (2.5%)	0.540 (0.085-3.428)	*p*=0.514
**Dominant model**				
GG	267 (79.7%)	65 (81.3%)	1.000 (reference)	
GA+AA	68 (20.3%)	15 (18.8%)	0.440 (0.051-3.772)	*p*=0.454
**SDF-1** **rs2839693**				
**Additive model**				
CC	263 (78.5%)	58 (72.5%)	1.000 (reference)	
CT	68 (20.3%)	22 (27.5%)	1.324 (0.345-5.083)	*p*=0.682
TT	4 (1.2%)	0 (0.0%)	---	---
**Dominant model**				
CC	263 (78.5%)	58 (72.5%)	1.000 (reference)	
CT+TT	72 (21.5%)	22 (27.5%)	1.311 (0.342-5.024)	*p*=0.693
**SDF-1** **rs266085**				
**Additive model**				
TT	119 (35.5%)	18 (22.5%)	1.000 (reference)	
TC	166 (49.6%)	47 (58.8%)	2.106 (1.090-4.069)	*p*=0.027
CC	50 (14.9%)	15 (18.7%)	2.208 (0.937-5.204)	*p*=0.070
**Dominant model**				
TT	119 (35.5%)	18 (22.5%)	1.000 (reference)	
TC+CC	216 (64.5%)	62 (77.5%)	2.130 (1.130-4.014)	*p*=0.019
**CXCR4** **rs2228014**				
**Additive model**				
CC	257 (76.7%)	63 (78.8%)	1.000 (reference)	
CT	70 (20.9%)	16 (20.0%)	1.103 (0.223-5.456)	*p*=0.905
TT	8 (2.4%)	1 (1.3%)	0.284 (0.029-2.786)	*p*=0.280
**Dominant model**				
CC	257 (76.7%)	63 (78.8%)	1.000 (reference)	
CT+TT	78 (23.3%)	17 (21.3%)	0.875 (0.184-4.165)	*p*=0.867
**CXCR4** **rs6430612**				
**Additive model**				
CC	310 (92.5%)	77 (96.3%)	1.000 (reference)	
CT	25 (7.5%)	3 (3.7%)	1.462 (0.132-16.189)	*p*=0.757
TT	0 (0.0%)	0 (0.0%)	---	*---*
**Dominant model**				
CC	310 (92.5%)	77 (96.3%)	1.000 (reference)	
CT+TT	25 (7.5%)	3 (3.7%)	1.462 (0.132-16.189)	*p*=0.757

The adjusted odds ratio (AOR) with their 95% confidence intervals were estimated by multiple logistic regression models.
